# Conditional Immortalization of Human B Cells by CD40 Ligation

**DOI:** 10.1371/journal.pone.0001464

**Published:** 2008-01-23

**Authors:** Martina Wiesner, Caroline Zentz, Christine Mayr, Rainer Wimmer, Wolfgang Hammerschmidt, Reinhard Zeidler, Andreas Moosmann

**Affiliations:** 1 Clinical Cooperative Group Molecular Oncology, GSF - National Research Center and Ludwig-Maximilians-Universität, Munich, Germany; 2 Institute for Human Genetics, Technische Universität, Munich, Germany; 3 Institute for Human Genetics, Ludwig-Maximilians-Universität, Munich, Germany; 4 Department of Gene Vectors, GSF - National Research Center for Environment and Health, Munich, Germany; 5 Department of Otorhinolaryngology, Ludwig-Maximilians-Universität, Munich, Germany; Oklahoma Medical Research Foundation, United States of America

## Abstract

It is generally assumed that human differentiated cells have a limited life-span and proliferation capacity *in vivo*, and that genetic modifications are a prerequisite for their immortalization *in vitro*. Here we readdress this issue, studying the long-term proliferation potential of human B cells. It was shown earlier that human B cells from peripheral blood of healthy donors can be efficiently induced to proliferate for up to ten weeks *in vitro* by stimulating their receptor CD40 in the presence of interleukin-4. When we applied the same stimuli under conditions of modified cell number and culture size, we were surprised to find that our treatment induced B cells to proliferate throughout an observation period of presently up to 1650 days, representing more than 370 population doublings, which suggested that these B cells were immortalized *in vitro*. Long-term CD40-stimulated B cell cultures could be established from most healthy adult human donors. These B cells had a constant phenotype, were free from Epstein-Barr virus, and remained dependent on CD40 ligation. They had constitutive telomerase activity and stabilized telomere length. Moreover, they were susceptible to activation by Toll-like receptor 9 ligands, and could be used to expand antigen-specific cytotoxic T cells *in vitro*. Our results indicate that human somatic cells can evade senescence and be conditionally immortalized by external stimulation only, without a requirement for genetic manipulation or oncoviral infection. Conditionally immortalized human B cells are a new tool for immunotherapy and studies of B cell oncogenesis, activation, and function.

## Introduction

It is believed that the proliferation capacity of normal differentiated human cells is limited *in vivo* and *in vitro*. The progressive shortening of telomeres – repetitive DNA sequences at the ends of the chromosomes – with each cell division ultimately leads to replicative senescence, characterized by a permanent growth arrest [1], [2]. Telomere shortening is counteracted by telomerase, which adds telomeric repeats to the chromosomes' ends and is expressed in germline cells, but can also be induced in certain somatic cells such as activated lymphocytes [3]–[6]. Cellular immortalization requires a mechanism to maintain telomeres and usually involves up-regulation of telomerase activity [1].

The requirements for immortalization of human cells *in vitro* are cell type–specific. Human embryonic stem cell clones constitutively display strong telomerase activity and are immortalized *in vitro* without requiring genetic manipulation [7]. For human fibroblasts and T lymphocytes, transduction with telomerase was necessary and sufficient to stabilize telomeres and to achieve immortalization in vitro [8]–[10]. Activation of T cells is associated with induction of endogenous telomerase activity [11], but at levels that appear to be insufficient to achieve their immortalization. For epithelial cells, the ectopic expression of telomerase was not sufficient for their immortalization; inactivation of the Rb/p16 pathway was additionally required [12]. The spontaneous *in vitro* immortalization of human somatic cells has so far been observed only in experiments with single human donors or in cells derived from patients with inherited genetic disorders predisposing to cancer [13], [14].

Activated human B lymphocytes display strong telomerase activity [3]–[6], associated not only with maintenance but with lengthening of telomeres after activation of B cells in germinal centers [4]. However, the possibility of very long-term proliferation or immortalization of normal human B cells *in vitro* has not been investigated so far, with the exception of B cells growth-transformed by the human oncogenic Epstein-Barr virus (EBV). EBV infection of normal human B cells generally results in the establishment of autonomously proliferating lymphoblastoid cell lines [15]. This process, though often ambiguously called “EBV immortalization”, produces cell lines that are mostly mortal and have low levels of telomerase activity [16], [17].

Alternatively, human B lymphocytes can be activated and induced to proliferate *in vitro* by triggering their surface receptor CD40 in the presence of interleukin-4 [18], a combination of signals mimicking B cell activation by T helper cells. CD40-stimulated B cell cultures have been used to model B cell differentiation to memory B cells or plasma cells *in vitro* [19]. Their potential to act as antigen-presenting cells to generate antigen-specific T cells in vitro for autologous immunotherapy was explored in detail [20], [21]. However, in these studies CD40/IL4 receptor-driven B cell cultures were not maintained for more than four to ten weeks [18], [20], [22]–[25]. After this period, the B cell cultures, apparently spontaneously, died out [18] or arrested and differentiated to plasmacytes [23]. The trigger of this cell death or differentiation, and the reason for the differences in differentation kinetics and maximum culture times of CD40-stimulated B cells reported by different investigators, have not been identified. Moreover, in studies that described relatively long-term culture of CD40-stimulated B cells (for up to ten weeks), donor-derived Epstein-Barr virus (EBV) was observed to pervade the cultures over time [18], [20], leaving open the question whether the establishment of strictly virus-free CD40-stimulated B cell cultures is possible with normal human adult donors, which are generally positive for EBV.

Here we describe that CD40-stimulated B cell cultures proliferate for vastly longer periods of time than previously reported when modified *in vitro* stimulation conditions are applied. We present evidence that such very long-term B cells can be established from a majority of healthy adult donors, have a constant phenotype representative of activated B cells, are free from EBV, remain dependent on regularly repeated CD40 ligand/IL-4 stimulation, and thus appear to be conditionally immortalized in vitro. These results suggest that an immortalization program intrinsic to a differentiated human cell type, the B cell, can be accessed by exogenous stimulation only.

## Results

### B cell stimulation conditions

**Figure 1:**
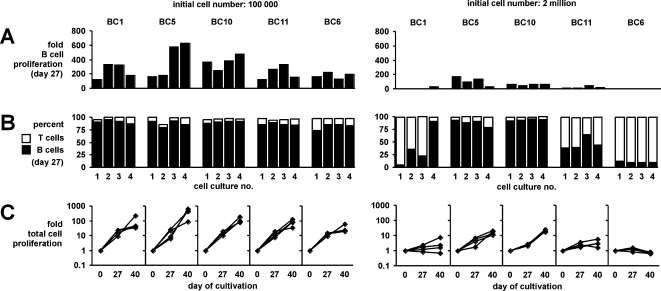
Conditions for the establishment of CD40-stimulated B cells. B cell cultures were established from 10^5^ PBMC (left) or 2×10^6^ PBMC (right). For each condition, four replicate cultures were set up from each of five donors. CD19+CD3– B cell and CD3+CD19– T cell content was assessed by flow cytometry. The increase in total B cell numbers (*A*), the proportions of B cells and T cells in the cultures on day 27 (*B*), and total increases in cell number on days 27 and 40 (*C*) were calculated.

We re-evaluated the conditions for CD40 stimulation of primary human B cells. In EBV carriers (>95% of the adult human population), one in about 10^4^–10^6^ B cells is EBV-infected. In an attempt to establish EBV-free CD40-stimulated B cell cultures, we plated unseparated peripheral blood mononuclear cells (PBMC) from EBV-positive donors in different numbers per microculture on CD40L-expressing stimulator cells [26] in the presence of interleukin-4 and cyclosporin A [20]. Cells were restimulated every 5 to 7 days with fresh stimulator cells and expanded when outgrowth became prominent. Surprisingly, we observed that rapid outgrowth of B cells was favoured if small initial cell numbers per culture were used (2×10^4^ to 5×10^5^ PBMC). After 27 days, cultures from five donors set up with 10^5^ PBMC had proliferated on average 282-fold with respect to B cells (Figure 1A) and were dominated by B cells (87±5% CD19+ cells, Figure 1B). With a higher initial cell number of 2×10^6^ PBMC, cell cultures proliferated more slowly, contained highly variable proportions of B cells (44±50%), were often dominated by T cells, and sometimes ceased to proliferate and died (Figure 1A,B). Cultures dominated by T cells were characterized by rapid destruction of the CD40L-expressing stimulator cell layer, as seen by microscopical examination (not shown). These results showed that the initial PBMC number per culture had to be kept low to facilitate rapid and efficient outgrowth of B cell cultures. At higher cell numbers, however, it appeared that T cells were activated in spite of the presence of cyclosporin A. These T cells might have hampered B cell expansion by elimination of stimulator cells or direct effects on B cells.

### Establishment of CD40-stimulated B cells

**Figure 2:**
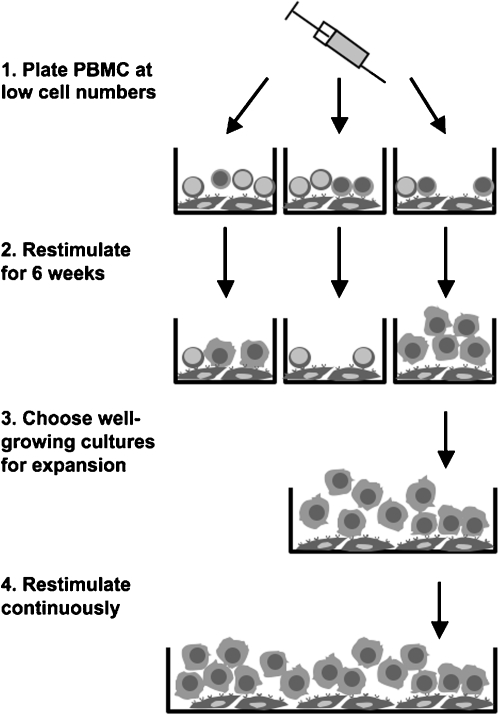
Overview of a protocol to establish long-term CD40-stimulated B cell cultures.

These observations led us to use a two-step protocol to establish long-term CD40-stimulated B cell lines from PBMC from various EBV-positive donors (Fig. 2). We set up individual microcultures by plating 20, 10, 5, or 2.5×10^4^ PBMC on CD40 stimulator cells in the presence of IL-4, and restimulated them every week. After about six weeks, we selected from each donor a well-proliferating culture derived from the lowest initial PBMC number that had led to robust outgrowth. This culture was further stimulated and expanded as before. At six weeks, 40–100% of replicate cultures set up with 10^5^ PBMC had given rise to proliferating B cell cultures, and in many donors proliferating cultures could also be obtained from 5 or 2.5×10^4 ^PBMC. Assuming that the PBMC contained 2 to 20% B cells, the founder populations of the B cell lines consisted of 250 to 20 000 B cells.

### Long-term maintenance of CD40-stimulated B cells

**Figure 3:**
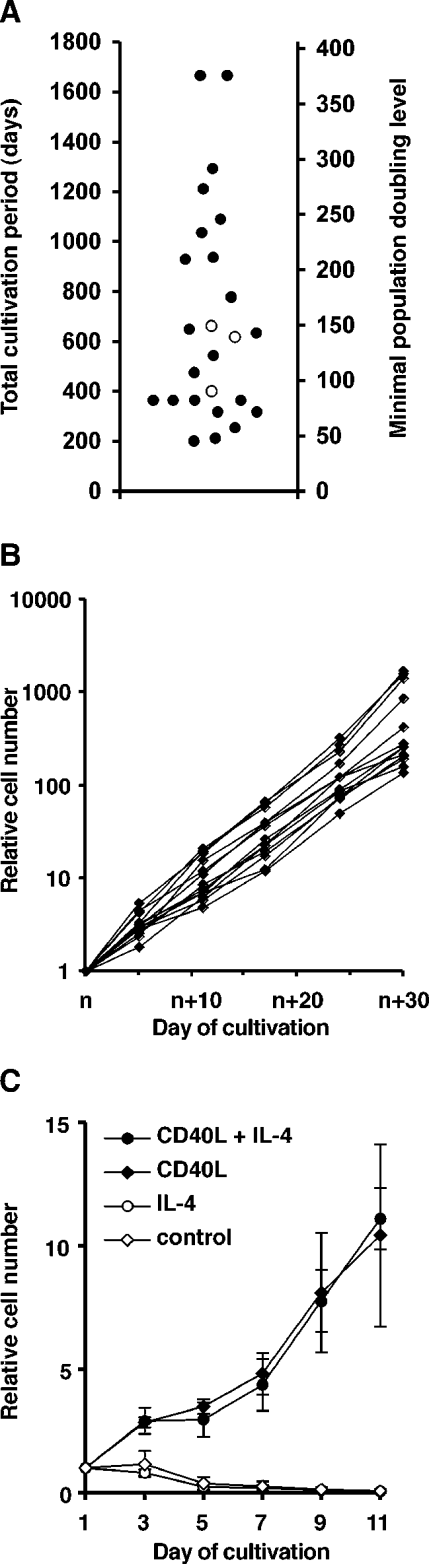
Long-term culture of CD40-stimulated B cell lines. (*A*) Culture periods and minimal proliferation doubling levels of Epstein-Barr virus–free CD40-stimulated B cell lines that were cultured for more than 200 days. Each cell line shown is from a different donor. Solid dots represent B cell lines that are currently proliferating in culture or were cryoconserved as proliferating cultures. Open dots indicate B cell lines that ceased to proliferate at the indicated time. (*B*) Proliferation of established CD40-stimulated B cell lines during a 30-day period. At the start of analysis (day n), B cell lines LXL5 and HXL7 were at day 884 of culture, the other B cell lines were at day 570, 512, 430, 251 (two B cell lines) or 155 (five B cell lines). (*C*) Dependence of B lymphoblast proliferation on CD40L and IL-4. B cells HXL7 or LXL5 (day 586 of culture) were grown for 11 days on CD40L-expressing murine fibroblasts or control fibroblasts in the presence or absence of IL-4, before cell counting and viability assessment by flow cytometry.

The majority of CD40-stimulated B cell cultures set up according to this protocol (Fig. 2) has continued to proliferate throughout the observation period. At present, CD40-stimulated B cells from 25 donors have been cultured, with continuous expansion, for more than 200 days (Fig. 3A). Cultures from eight donors have proliferated for more than 900 days, representing more than 200 proliferation doublings. In three instances, we observed that CD40-stimulated B cell cultures ceased to proliferate, which occurred after 400 to 660 days (Fig. 3A). In the other 22 cultures, we did not observe a decrease in proliferation or any indication of senescence or crisis. The proliferation of lymphocyte cultures over more than 170 or 180 doublings has been interpreted to indicate cellular immortalization [10], [16]. By analogy, our results suggest that stimulation of B cells with CD40L/IL-4 may induce a state of immortalization, and that this is true for B cell cultures from a majority of healthy adult donors.

In early as well as in advanced stages of culture, established CD40-stimulated B cell cultures multiplied 100 to 1000-fold every 30 days (Figure 3B). B cell proliferation in established cultures remained strictly dependent on continuous stimulation by CD40L, but was independent of IL-4 over an 11-day interval after CD40 stimulation (Figure 3C). If CD40-stimulated B cells were cultured by CD40 stimulation in the absence of IL-4 for prolonged periods, however, proliferation gradually slowed down and ceased after about 4 to 8 weeks (not shown).

**Figure 4:**
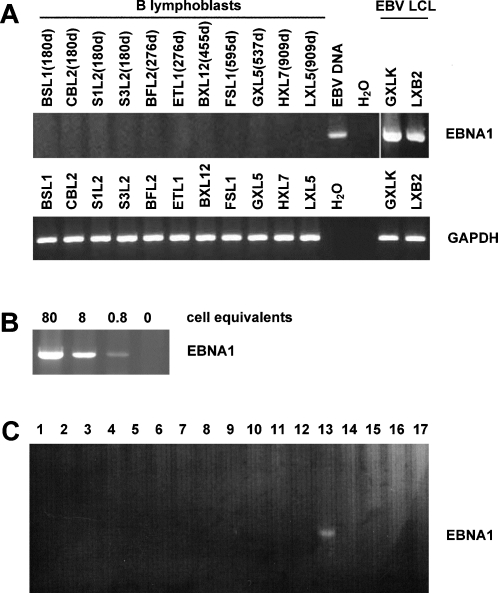
Long-term CD40-stimulated B cells remain EBV-free. (*A*) PCR was used to detect the EBV EBNA1 gene in CD40-stimulated B cells from eleven donors after various culture periods (the number of days is indicated in parentheses for each cell line). EBV DNA and EBV-infected B lymphoblastoid cell lines GXLK and LXB2 were used as positive controls. (*B*) The PCR protocol allowed the detection of one EBV-infected cell equivalent per reaction. (*C*) In this example of a routine PCR analysis of early-passage CD40-stimulated B cell cultures, EBV was detected in one of 17 cultures from three different EBV-positive donors. B cell cultures had been set up with 100,000 PBMC each and were tested on day 80–90 of culture.

All CD40-stimulated B cell lines were regularly tested for the presence of EBV DNA by PCR and found to be negative in the majority of cases (Figure 4). Among all cultures expanded from our 25 donors, we identified two EBV-positive B cell lines; in both of these, EBV became detectable before day 100 of culture. In both cases, another B cell culture from the same donor was available, which was EBV-negative. Only CD40-stimulated B cell cultures that were shown to be EBV-negative were included in Figure 3 and subsequent analyses. Of our 25 donors, 24 were seropositive for EBV, only one was seronegative. Accordingly, these results indicate that the routine generation of EBV-free, conditionally immortalized CD40-stimulated B cell cultures is possible, in the first attempt, with >90% of EBV-positive healthy adult donors.

### Constant immunophenotype of CD40 B cells

**Figure 5:**
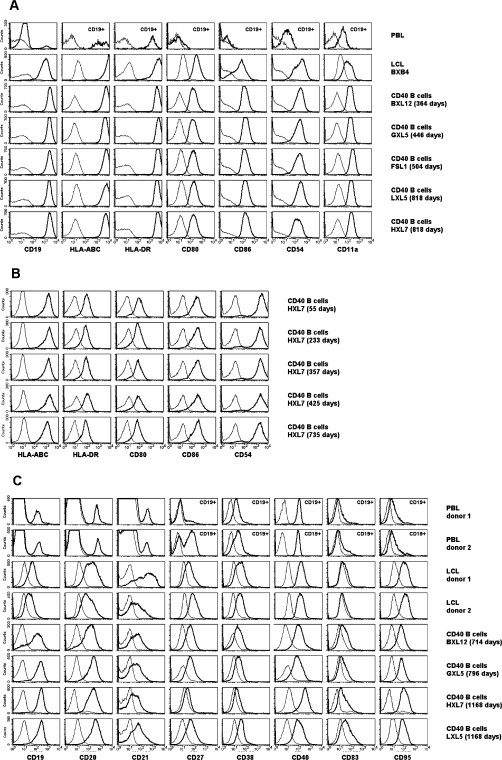
Phenotype of long-term CD40-stimulated B cells. (*A*) Five CD40-stimulated B cell lines from different donors after 364 to 818 days of culture were stained for CD19, HLA-ABC and HLA-DR, the costimulatory molecules CD80 and CD86, and the adhesion molecules CD54 and CD11a, and were analyzed by flow cytometry. Thick lines represent staining with specific antibody, thin lines with a matched isotype control. Peripheral blood lymphocytes gated on forward and sideward scatter (top left diagram) or additionally gated for CD19 expression (top row, marked with an asterisk) and an EBV-infected B lymphoblastoid cell line, BXB4, were used for comparison. (*B*) One CD40-stimulated B cell line, HXL7, was stained for several of the above markers after various periods of culture, ranging from 55 to 735 days. (*C*) CD40-stimulated B cells, EBV-transformed B lymphoblastoid cell lines, and PBL were analyzed for surface expression of a panel of B cell differentiation and activation markers. Specific stainings and controls are represented as in (*A*).

We investigated the phenotype of long-term CD40-stimulated B cells. Consistent with previous results [20], [27], we observed a uniform pattern of high expression of antigen-presenting molecules (HLA-ABC and HLA-DR), costimulatory molecules (CD80 and CD86) and adhesion molecules (CD11a and CD54; Figure 5A), reflecting the role of activated B cells in T cell activation. Expression levels remained constant during long-term culture (Figure 5B) and were similar to levels detected on EBV-transformed B lymphoblastoid cell lines (Figure 5A). In addition, CD40-stimulated B cells robustly expressed the B cell markers CD19, CD20, CD21, the activatory receptor CD40, and the apoptotic signal receptor CD95 (Fig. 5C). Most CD40 B cell cultures also expressed the memory B cell marker CD27. Their staining levels for the differentiation marker CD38 were similar to *ex vivo* peripheral B cells, suggesting the absence of detectable plasma cell differentiation. Expression of CD83, which is associated with B and T cell activation and dendritic cell maturation, was elevated in comparison to *ex vivo* peripheral B cells or EBV-activated B cells (Fig. 5C). Overall, long-term CD40-stimulated B cells continously displayed a phenotype characteristic for activated B cells; other cell types were not found in the cultures.

### Clonal and cytogenetic analysis

**Figure 6:**
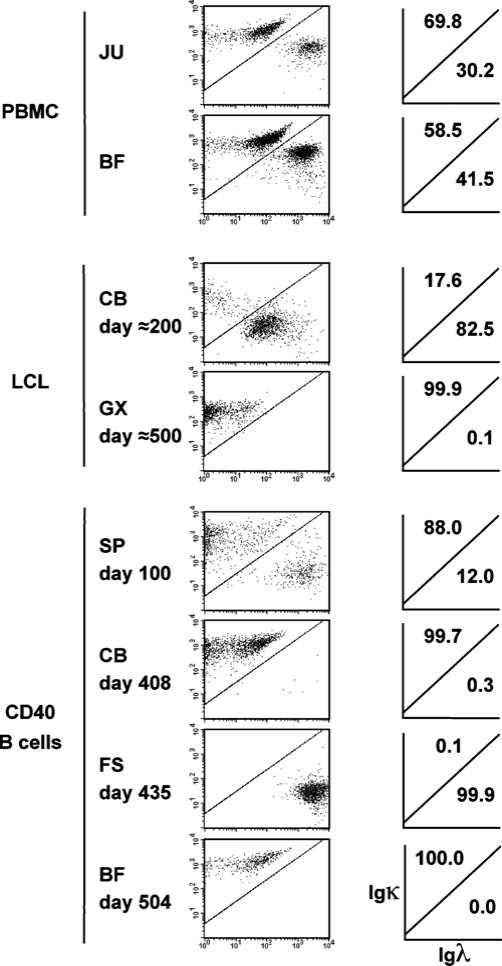
Immunoglobulin light chain expression patterns. CD19+ PBMCs *ex vivo*, EBV-transformed B lymphoblastoid cell lines, and CD40-stimulated B cells from various different donors were cultured for the indicated periods of time and analyzed for expression of Igκ and Igλ chains by flow cytometry.

To obtain information about the clonal diversity of long-term CD40-stimulated B cells, we stained for expression of immunoglobulin κ or λ light chains (Figure 6). B cells from PBMCs ex vivo, as well as EBV LCLs or CD40-activated B cell cultures in earlier passage, were a mixture of cells expressing either the κ or λ isoform. After 400 days of culture, however, each CD40 B cell culture or LCL nearly completely (>99.5%) expressed only κ or only λ light chains, suggesting monoclonality or a low degree of oligoclonality.

A selection of long-term CD40-stimulated B cells was analyzed for chromosomal integrity (Table 1). Three of nine cell lines contained only cells with a normal karyotype. Two further cell lines contained both normal cells and cells carrying an additional copy of chromosome 2; another cell line homogeneously displayed trisomy 2. The remaining three cell lines had chromosomal aberrations involving chromosome breakages or recombinations. Consistent with the proposition that trisomy 2 is a marker of senescence rather than transformation [28], [29], the B cell line that had trisomy 2 in all cells was the only one in this panel that later ceased to proliferate (see also Figure 3A).

**Table 1:** Karyotypes of CD40-stimulated B cell lines

B cell line	Day of analysis	Karyotype
AHL	461	46,XY
GXL5	611	46,XY
BXL12	1051	46,XX
BSL	831	46,XX [7]
		47,XX,+2 [15]
f46L	931	46,XX [21]
		47,XX,+2 [3]
BFL2*	350	47,XX,+2
LXL5	768	46,XY,t(14;18)
HXL7	776	46,XY,der(4)t(2;4)
RZL	748	46,XYqS,der(14),der(19)

Cell line BFL2 (*) ceased to proliferate near day 400 (see Figure 3A). All other cell lines represented here maintained their proliferation during the observation period. At least 15 cells were examined per B cell line. Numbers in square brackets [] indicate the number of metaphases evaluated.

### Telomere stabilization and telomerase activity

**Figure 7:**
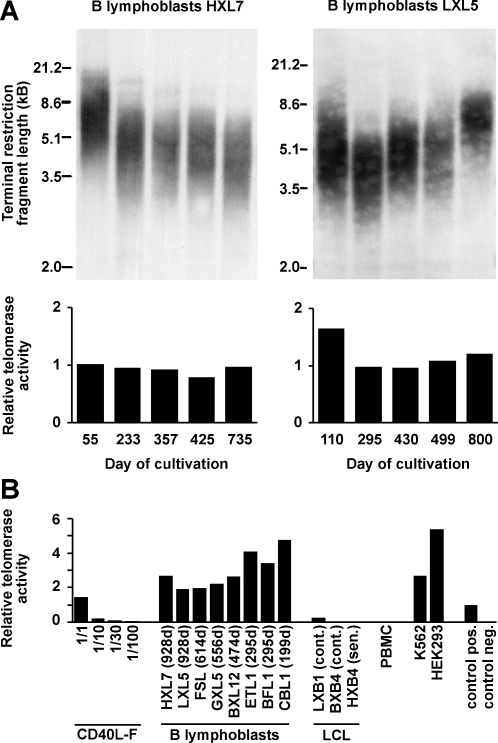
Telomere length and telomerase activity in long-term CD40-stimulated B cells. (*A*) Telomeric terminal restriction fragment (TRF) length and telomerase activity in two long-term CD40 B lymphoblast lines, HXL7 and LXL5, over time. Telomere-containing terminal restriction fragments were obtained by restriction hydrolysis of genomic DNA. Telomerase activity was semiquantitatively determined according to a modified telomeric repeat amplification protocol (TRAP)[50] in an ELISA format. An internal PCR standard template was co-amplified in each reaction and detected with an independent probe. As a positive control, a template containing 8 telomeric repeats was PCR-amplified and analyzed in the same manner. Relative telomerase activities of samples with respect to the positive control were calculated. (*B*) Telomerase activities of various CD40-stimulated B lymphoblast lines. Relative telomerase activities of CD40 B lymphoblasts were in a similar range as standard immortalized cell lines (K562 and HEK293) and the artificial telomere product used as positive control. The maximum number of CD40L-expressing feeder cells that might have been present in the CD40 B lymphoblast preparations, corresponding to a dilution of 1/30, had negligible telomerase activity.

We assessed telomere length and telomerase activity during long-term culture of two CD40-stimulated B cell lines (Figure 7A). Average telomere length stabilized at above 3.5 kilobase pairs (kbp) in one B cell line, HXL7, for the 735-day period covered by the analysis. In another B cell line, LXL5, telomere length reached a minimum of about 4 kbp at day 295, then increased again. Both B cell lines showed high levels of telomerase activity throughout the time of this analysis (735 or 800 days, respectively). Telomerase activities of eight CD40-stimulated B cell lines at various times of culture, and of the transformed permanent cell lines HEK293 and K562, were in the same order of magnitude (Figure 7B). Notably, telomerase activity in EBV-transformed B lymphoblastoid cell lines, either in the proliferative phase or in senescence/crisis, was much lower (Figure 7B), highlighting that telomerase is much more strongly activated by CD40/IL-4 stimulation than by EBV transformation. Thus, while telomerase-dependent immortalization is an exception in EBV-transformed B cells [16], [17], it appears to be the rule in CD40-stimulated B cells.

### Stimulation with oligonucleotides

**Figure 8:**
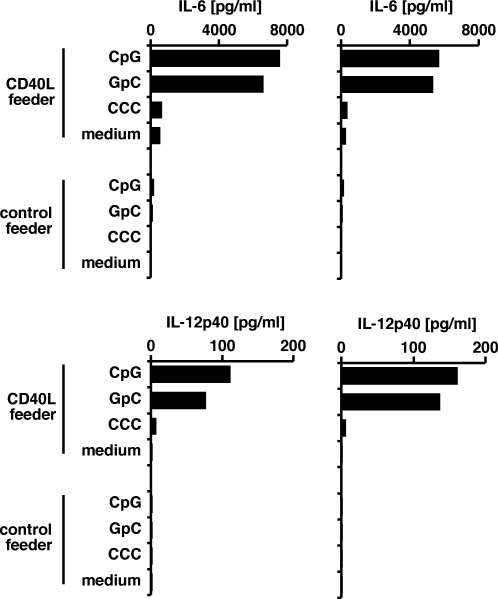
Long-term CD40-stimulated B cells are activated by oligodeoxynucleotides. Long-term CD40 B cells were treated with TLR9-agonistic oligonucleotides in the presence of CD40L-expressing or non-expressing fibroblast feeder cells. After 4 days, secretion of IL-6 and IL-12p40 was analyzed in an ELISA. TLR9 stimuli and controls included a phosporothioate oligonucleotide (ODN 2006) containing 4 CpG motifs (“CpG”), an oligonucleotide of the same sequence except that each CpG dinucleotide was replaced by a GpC dinucleotide (“GpC”), an oligonucleotide composed of twenty phosphorothioate deoxycytidines (“CCC”), and a medium-alone control. For each condition, two CD40 B cell cultures from different donors were tested. At the time of the experiment, CD40 B cells had been cultured for 570 days (BXL12, top left), 652 days (GXL5, top left), 1074 days (line LXL5, bottom left), or 702 days (line GXL5, bottom right).

We investigated whether long-term cultured CD40-stimulated B cells can be used as models to study selected aspects of B cell activation. The stimulation of Toll-like receptor 9 (TLR9) by CpG-containing oligonucleotides in combination with CD40 stimulation induces primary human B cells to produce interleukin-6 and interleukin-12 [30]. We tested whether long-term CD40-stimulated B cells are still susceptible to this type of activation. Indeed, we observed that the CpG-containing phosphorothioate deoxyoligonucleotide 2006 [31], if combined with CD40 stimulation, induced long-term B cell lines to secrete interleukin-6 and interleukin-12p40 (Figure 8). Consistent with earlier observations [32], [33], a phosphorothioate deoxyoligonucleotide with the CpG motifs replaced by GpC motifs led to similar levels of cytokine secretion. In contrast, a control oligonucleotide carrying only cytosines had no effect. Thus, a reactivity pattern of primary B cells against stimulatory ligands is retained in conditionally immortalized CD40-stimulated B cells. Considering these B cells are available in unlimited amounts and are not contaminated by other types of immune cells, they may serve as useful tools in studies of B cell reactivity.

### Expansion of antigen-specific T cells

**Figure 9:**
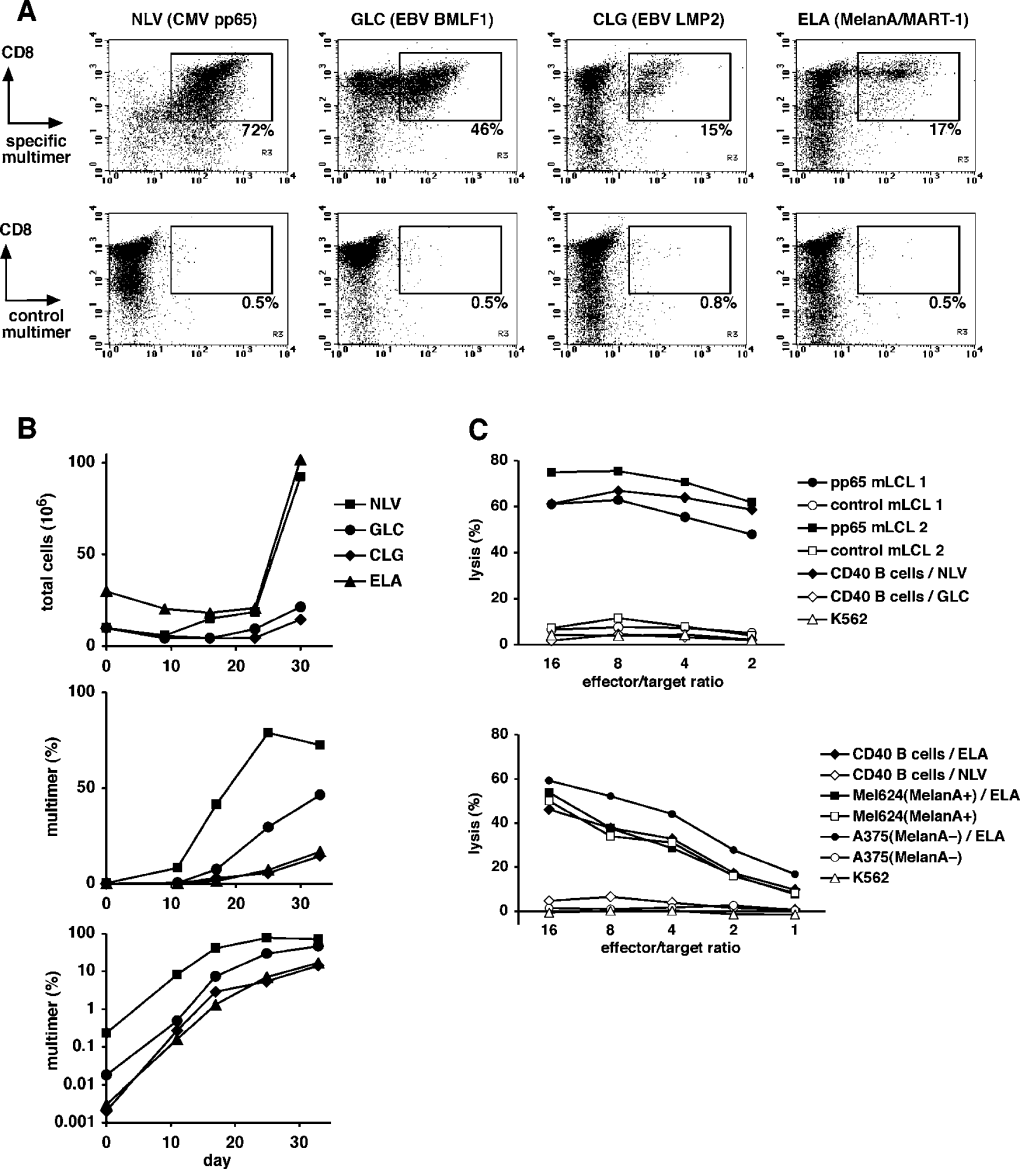
Specific expansion of cytotoxic T cells by stimulation with long-term CD40-stimulated B cells. (A) Frequency of antigen-specific CD8+ T cells in four T cell lines after 33 days of repeated stimulation with peptide-loaded autologous long-term CD40 B cells. B cells had been cultured for 693 days at the beginning of the T cell stimulation. Either of four HLA-A2-restricted antigenic peptides derived from viral or melanoma antigens (abbreviated NLV, GLC, CLG, and ELA) was used for stimulation. The frequency of specific T cells was assessed by staining with the corresponding specific HLA/peptide multimer reagent. For control stainings, an HLA/peptide multimer containing an irrelevant peptide was used for staining (lower row): ELA for NLV-stimulated cultures and NLV for all other cultures. (B) Expansion of antigen-specific T cells after stimulation with peptide-loaded long-term CD40 B cells. Total cell numbers (top) in the T cell cultures were determined by microscopic counting. The proportion of antigen-specific CD8+ T cells, represented in linear (middle) and logarithmic scale (bottom), was determined by specific HLA/peptide multimer staining at various times of T cell culture. (C) The cytotoxic reactivity of two of these T cell cultures against cells endogenously presenting the target antigen was assessed in a calcein release assay. Top: T cells expanded with the peptide NLV (from CMV pp65) were tested against HLA-A2-matched mini-LCLs endogenously expressing pp65 or not, autologous CD40 B cells loaded with the target peptide NLV or the control peptide GLC, or K562 cells to test for natural killer-like reactivity. Bottom: T cells expanded with the peptide ELA from MelanA were tested against HLA-A2-matched melanoma cell lines either expressing MelanA (Mel624) or not (A375) that had either been additionally loaded with the ELA peptide or not. Further targets included autologous CD40 B cells loaded with the target peptide ELA or the control peptide NLV, and K562 cells.

Previous studies had shown that short-term CD40-stimulated B cells loaded with antigenic peptides efficiently stimulate and expand antigen-specific T cells [25], [34]. To verify that long-term CD40-stimulated B cells were similarly efficient in this application, we expanded T cells from an HLA-A2 positive, CMV/EBV-seropositive donor with peptide-loaded B cells from an autologous, 693-day old CD40-stimulated B cell line. To compare how efficiently T cells with high and low frequency could be expanded from the T cell repertoire of this donor, we used HLA-A2-restricted peptide epitopes from four different antigens: the CMV tegument protein pp65, the BMLF1 gene product and the latent membrane protein 2 (LMP2) of EBV, and the melanocyte/melanoma antigen MelanA/MART-1. According to HLA/peptide multimer staining, the frequencies of T cells specific for these epitopes spanned two orders of magnitude: CD8+ T cells specific for pp65 (peptide epitope NLV) were present in PBMC at a high frequency of 0.23%, CD8+ T cells specific for the BMLF1 epitope (GLC) were ten times less frequent (0.018%), and CD8+ T cells specific for LMP2 (epitope CLG) and MelanA (epitope ELA) were rare in this donor's repertoire (0.002 and 0.003% of PBMC). When PBMC were stimulated with CD40 B cells loaded with one of these four antigenic peptides in four separate cultures, T cells specific for each peptide were specifically expanded with similar efficiency, resulting in a 10–100 fold enrichment during the first 11 days and a 100–1000 fold enrichment during the first 17 days (Figure 9B, lower panel). After 33 days of expansion, 17–72% of total cells in the cultures were antigen-specific CD8+ T cells (Figure 9A and B), and antigen-specific T cells had been expanded between 2.900 (CMV pp65) and 19.000 fold (MelanA). The observation that MelanA-specific T cells could be expanded at least as well as T cells recognizing viral antigens is noteworthy considering that the virus-specific T cells were derived from the central or effector memory T cell pool, while MelanA-specific T cells are expected to be an expanded population of T cells with a naive phenotype in healthy donors [35], and therefore the requirements for their activation might be more stringent.

To test whether these CD8+ T cells were functionally competent, we assessed the cytotoxic reactivity of the T cell lines specific for the viral antigen CMV pp65 and the tumor/autoantigen MelanA. We found that these T cells efficiently recognized HLA-A2+ target cells that endogenously expressed the target antigen from which the epitope was derived (Figure 9C). The pp65-specific T cells were tested against pp65-expressing mini-lymphoblastoid cell lines (pp65 mini-LCLs), B cells that are growth-transformed by stably carrying a mini-EBV genome that also constitutively expresses CMV pp65 in the cells [36]. HLA-A2-matched pp65 mini-LCLs were recognized and lysed by the T cells with nearly equal efficiency as the autologous CD40 B cells loaded with the pp65 epitope peptide NLV. Control mini-LCLs (pp65-negative) or CD40 B cells loaded with control peptide were not lysed. MelanA-specific T cells were tested for cytotoxicity against MelanA-expressing or non-expressing, HLA-A2-matched melanoma cells. A melanoma cell line endogenously expressing MelanA, Mel624-38, was recognized at similar levels as the same cell line when additionally loaded with the target peptide ELA or as CD40 B cells loaded with ELA peptide. MelanA-negative melanoma cells, however, were only recognized when exogenously loaded with ELA peptide.

Altogether, these results provide an example that antigen-specific CD8+ T cell populations of high or low frequency in the periphery can efficiently be expanded by stimulation with long-term CD40-stimulated B cells, and acquire or maintain antigen-specific cytolytic function after expansion.

## Discussion

Here we describe that peripheral human B cells from healthy donors were capable of very extended proliferation after specific external activation, justifying these cells' description as being conditionally immortalized. By CD40L/IL-4 stimulation, B cells were maintained in a proliferative and activated state characterized by the expression of activation markers and the absence of telomere erosion. They proliferated beyond the 170–180 population doublings that have been proposed to indicate immortalization of lymphocytes [10], [16]. Their long-term proliferation remained dependent on the signals provided by exogenous CD40L and IL-4, both in early and late passage. Therefore, the state of these cells bears a much closer resemblance to physiological T-cell driven clonal expansion than to malignant transformation.

**Figure 10:**
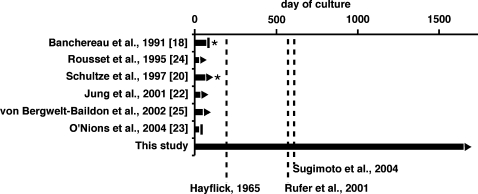
Culture periods of CD40/IL-4-stimulated B cells reported in different studies. For each study, the longest period during which B cell proliferation was observed is represented. A vertical bar indicates that proliferation was observed to terminate. A triangle indicates that the observation period ended at a time when B cells were still profilerating. An asterisk marks studies in which EBV infection of late-passage CD40/IL-4-stimulated B cell cultures was reported as a typical outcome of B cell cultivation, or was positively observed without information given about the frequency of such an event. Vertical dashed lines indicate proposed limits to the proliferation of non-immortalized human fibroblasts [39], T cells [10] or EBV-transformed B cells [16], using an estimation that these cells divide twice a week on average.

The CD40/IL-4 cultivation system for human B cells was first described many years ago [18]. Similar CD40 systems have since been used for investigations in various fields like B cell differentiation, immunoglobulin production and T cell activation, documented in many studies of which we can only mention same examples [19], [20], [22]–[26], [37], [38]. It seems therefore remarkable that the generation of conditionally immortalized B cell lines with a CD40 system has, to our knowledge, not been described up to now. Figure 10 visualizes the fact that in previous studies CD40-activated B cells were generally cultivated for about 70 days or less. Therefore, they had undergone much less proliferation doublings than required to cross the limits to proliferation that were described for human cells that are mortal in vitro, like fibroblasts [39], CD8+ T cells [10], or EBV-transformed B cells [16].

Previously, CD40/IL-4-activated B cell cultures, in the hands of different investigators, could have different fates. In their initial study [18], Banchereau et al. described two phenomena interfering with CD40/IL4-driven B cell outgrowth: the spread of EBV in the cultures in later passage, and an unexplained “dying out” of the cultures before 10 weeks of cultivation. O'Nions and Allday [23] observed that CD40-activated B cells ceased to proliferate even earlier, associated with their differentiation to plasma cells [23]. Schultze et al. [20] cultivated CD40-activated B cells for up to 65 days without observing B cell differentiation or a decrease in proliferation, but again EBV infection was found in an unspecified proportion of the cultures. The authors of the latter study, in contrast to others, also used cyclosporin A in their culture protocol, which might have been an important factor in achieving prolonged B cell proliferation, and it appears possible that they might have achieved unlimited CD40/IL-4-dependent proliferation using their protocol, provided EBV could have been eliminated.

We suppose that the generation of conditionally immortalized B cells in this study was made possible due to (a) the use of cyclosporin A [20], reducing the in vitro reactivation of T cells, (b) the elimination of EBV by using small numbers of 100,000 PBMCs or less, containing 20,000 B cells or less, to initiate each culture, and (c) a reduction of the absolute number of T cells introduced into each culture (<90,000), again by using small numbers of unpurified PBMCs per culture. To our knowledge, none of the previous studies combined all of these three aspects. For example, Banchereau et al. [18] used 10^5^ purified B cells per culture (>97% purity) in the absence of cyclosporin A. Compared to our protocol, less T cells were introduced per culture, but the risk of reactivating them was raised, and more total B cells were used per culture, raising the risk of introducing EBV. Similar considerations apply for the other studies. It is likely that the inhibition and elimination of T cells in such a culture system is crucial because both B cell-specific and, if EBV is present, EBV-specific T cells might have a role in preventing outgrowth, in analogy to the situation in EBV-mediated B cell outgrowth [40].

We observed that conditionally immortalized CD40-activated B cells stabilized or re-increased their telomere length over time. Upon stimulation *in vivo* in germinal centers, human B cells can elongate their telomeres even beyond their length in naive B cells [4]. Telomere elongation can also be a result of *in vitro* B cell stimulation [41], and the maintenance of telomerase activity in activated B cells for several weeks has been described [22]. Still, on average, the telomeres of B cells, as well as those of T cells, are becoming shorter during a human lifetime [11], suggesting that endogenous telomerase cannot globally compensate for telomere loss during extended proliferation *in vivo* [42]. Our results show, however, that *in vitro* a subset of B cells may fully compensate for telomere loss during extended periods of time if a sufficiently strong exogenous stimulus is regularly provided. Specific immune responses depend on extensive clonal expansion of specific T or B lymphocytes, and telomerase is very probably critical in securing immune function by maintaining the lymphocytes' replicative potential [43]. Notably, although T cell activation is coupled to telomerase activation, telomeres of T cells inevitably shorten after their primary activation [44], and to achieve immortalization of T cells *in vitro* it was generally necessary and sufficient to ectopically express telomerase [9], [10]. Consistent with previous reports, our data suggest that the situation in human B cells is different. At least a subset of them appears to be equipped with an immortalization program that ensures telomere stabilization and can be accessed and maintained by applying extracellular ligands only. Such a difference in the proliferative potential of B and T cells might not be surprising, because B cells, having to undergo somatic hypermutation accompanied by heavy selection of affinity-matured cells, probably require an even greater proliferative potential than T cells.

Our cytogenetic analyses showed that a majority of long-term CD40-stimulated B cell lines contained cells with an intact karyotype, either exclusively or to a significant proportion. This situation is in marked contrast to that found in EBV-transformed B-lymphoblastoid cell lines (LCLs), where long-term growth and immortalization is necessarily associated with the acquisition of chromosomal aberrations, usually in combinations more complex than observed in any of the CD40-stimulated B cell lines analyzed by us [45]. Moreover, the necessity in LCLs to select for rare mutated cells capable of further proliferation often manifests itself in the form of a proliferative crisis, a phenomenon we did not observe in CD40-stimulated B cell lines. In this context, it is noteworthy that three of our B cell lines contained cells with an additional copy of chromosome 2, an aberration that has been associated both with in vitro senescence of human T cells from old-age donors [29] and with human malignancies [46]. One of our B cell lines homogeneously showed trisomy 2, and this line later underwent senescence. It is an interesting question whether the occurrence of trisomy 2 generally predicts senescence in this culture system. Taken together, although it was not possible to generate chromosomally intact long-term CD40-stimulated B cells from each normal donor, they could be obtained from several donors, and the level of genetic stability of this type of B cell culture appeared to be generally higher than that observed in LCLs and no lower than that of other cells that are cultured in bulk for extended periods, for example human embryonic stem cells [47]. Thus, our data seem to indicate that the acquisition of genetic abnormalities is not required for the conditional immortalization of human B cells in this system.

We provide two examples of possible applications of long-term CD40-stimulated B cells. In our first example, we showed that they strongly and specifically react to stimulatory oligodeoxynucleotides with the secretion of cytokines. Studies on the activation of human B cells by innate immune receptor ligands usually rely on the use of primary B cells, which need to be purified in complicated procedures and are only available in limited numbers and in purities well below 100%. However, a small contaminating cell population can significantly disturb such experiments. The availability of normal CD40-activated B cell lines in unlimited numbers and essentially 100% purity could greatly facilitate such studies in the future.

In our second application of conditionally immortalized CD40-activated B cells, we showed that they can be used to specifically (re-)activate and expand antigen-specific CD8+ T cells in vitro. Dominant as well as subdominant memory T cell populations, and T cells that were likely a part of the naive repertoire, could equally well be expanded in vitro using peptide-loaded long-term CD40-activated B cells. Previous studies have shown in detail that shorter-term CD40-activated B cells can efficiently activate and expand populations of frequent or rare memory T cells and naive T cells [21], [25], [34], [48], [49]. Here we provide evidence that conditionally immortalized B cells can be used to the same purpose.

To our knowledge, CD40-stimulated B cells are the first example of a differentiated cell type from healthy human donors that undergoes conditional immortalization *in vitro,* in the absence of genetic manipulation. Their conditional immortalization was achieved by external stimulation with analogs of physiological ligands. Thus, human B cells display what was previously thought to be a unique feature of human stem cells and neoplastic cells. The future will show whether non-invasive mechanisms will be identified to break the proliferation limit of other human differentiated non-neoplastic cell types, or whether it will emerge that B cells have singular proliferation properties that are rooted in their unique biological function. It will also be important to investigate if and how individual B cell subsets differ in their capacity to undergo immortalization. However that may be, conditionally immortalized CD40-stimulated B cells present new opportunities to study B cell function and differentiation, and they offer themselves as tools for individualized cell or gene banking and for use in cellular immunotherapy.

## Materials and Methods

Standard medium was RPMI-1640 with 10% fetal calf serum, penicillin (100 U/ml), streptomycin (100 µg/ml), and sodium selenite (100 nM). For CD40-stimulated B cell culture, recombinant human interleukin-4 (IL-4; 2 ng/ml; R&D Systems) and cyclosporin A (CsA; 1 µg/ml; Novartis) were added.

### CD40-stimulated B cell cultures

CD40-stimulated B cell cultures were generated by co-culture of PBMC with murine fibroblastic L cells stably transfected with the human CD40 ligand gene [26]. To prepare stimulator cell plates, CD40L-expressing L cells were irradiated (100 Gy) and plated at 0.7–1.0×10^6^ cells per 12-well or 96-well plate. Plates were used for B cell stimulation 1 to 9 days later.

For comparisons of CD40-stimulated B cell outgrowth from different cell numbers in short-term assays (Figure 1), PBMCs were plated onto a layer of irradiated CD40L-expressing L cells at approximately constant cell per volume and cell per surface area ratios, i.e. 100,000 PBMC per well of a 96-well plate (0,33 cm^2^) in 200 µl, or 2 million PBMC distributed to two wells of a 12-well plate (2×3.5 cm^2^) in 2×1 ml of medium containing IL-4 and CsA. In subsequent stimulations (every 5–7 days), those wells that counted as one culture were pooled, and the cells were replated on new stimulator cells at the same conditions. When cell outgrowth occurred (appearance of cell clumps and acidification of medium), cells were expanded twofold per stimulation.

To set up long-term CD40-stimulated B cell cultures, peripheral blood mononuclear cells (PBMC) were distributed to 96-well stimulator cell-containing plates at 2.5, 5, 10 or 20×10^4^ cells per well in 200 µl of medium containing IL-4 and CsA. Four to eight cultures were set up for each cell number. Every 5–7 days, cells were transferred to new stimulator cell plates. Densely grown B cell cultures were expanded two- to threefold with respect to area and volume, less well-grown cultures were transferred without expansion. Depending on the donor, outgrowth occurred after 1–4 weeks, with varying intensity in cultures derived from different input PBMC numbers. After 6 weeks, one to four well-growing cultures derived from the lowest applicable input PBMC number (usually 2.5 or 5×10^4^ cells per well) were chosen for further expansion. Established CD40-stimulated B cell cultures were maintained in 12-well stimulator cell-coated plates. Cultures were replated every 5 to 7 days, expanding 3 to 4-fold. Maximal cell densities of B cell cultures were 0.8–2×10^6^ cells/ml.

### Flow cytometry

The following fluorescent dye–conjugated antibodies were used: CD3–PECy5, CD4-FITC, CD8–APC, CD19–PE, CD21–APC, CD54–APC, CD80–FITC, CD83-FITC, CD86–APC, HLA-DR–PerCP (Becton-Dickinson), HLA-ABC–FITC (Acris), CD11a–FITC, CD38–PE, CD95–FITC (ImmunoTools), CD20–FITC, CD27-PE, CD40–PE (BioLegend), Ig light chains kappa-FITC/lambda-PE (Dako). PBMC, B cell, or T cell populations were gated using forward and side scatter. For proliferation/viability analysis, samples from resuspended cell cultures, APC-labeled fluorescent beads in standardized numbers (10 000 beads per sample; Becton-Dickinson), and the dye TO-PRO3 (5 µM; Molecular Probes) were added together and immediately analyzed by flow cytometry. Forward/side scatter–gated cells negative for TO-PRO3 staining were interpreted as viable cells.

For HLA/peptide multimer staining of antigen-specific T cells, we used PE-labeled HLA-A2/peptide tetramers (“iTAg”, Beckman Coulter) presenting the CMV pp65 peptide NLV (NLVPMVATV) or the EBV BMLF1 peptide GLC (GLCTLVAML), and PE-labeled HLA-A2/peptide pentamers (“Pro5”, Proimmune, Oxford, UK) presenting the EBV LMP2 peptide CLG (CLGGLLTMV) or the mutated MelanA peptide ELA (ELAGIGILTV). PBMC or cultured T cells were stained with an empirically determined amount of HLA/peptide multimer for 15 minutes a room temperature, washed, and counterstained with CD4-FITC, CD3-PECy5, and CD8-APC antibodies. In PBMC, unspecific background of apparently CD8+ multimer+ cells was below 1/200,000 cells.

### Telomere length analysis

Telomere length was analyzed using the “TeloTAGGG” Telomere Length Assay kit (Roche). Cellular DNA (2 µg) was digested with *Hinf*I and *Rsa*I, separated by agarose gel electrophoresis, transferred to a nylon membrane, and hybridized with a digoxigenin-labeled telomere-specific probe.

### Telomerase activity

Telomerase activity was assessed using a semi-quantitative modification (Telomerase PCR ELISAplus, Roche, Mannheim, Germany) of the TRAP assay [50]. Telomerase-containing cell lysates (from 3000 cells per reaction) were used to elongate an artificial telomerase substrate oligodesoxynucleotide. Elongation products and an alternative template DNA (internal standard) were co-amplified by PCR. PCR-amplified telomerase products and internal standard products were separately quantified in an ELISA format using complementary digoxigenin-labeled DNA probes. As positive control, 10^−19^ mol of a telomerase product analog containing 8 telomeric repeats was co-amplified with the internal standard. Relative telomerase activity (RTA) with respect to the positive control was calculated according to
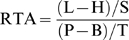
from the following absorption values: L, cell lysate; H, heat-inactivated cell lysate; S, internal standard of cell lysate; P, positive control; B, cell lysis buffer only; T, internal standard of positive control.

### Polymerase chain reaction

EBV DNA was detected by amplification of a 682-bp fragment from the *EBNA1* gene, using primers 5′- CCA GTA GTC AGT CAT CAT CAT CCG -3′ and 5′- TGG AAA CCA GGG AGG CAA ATC -3′. Per reaction, 400 ng of total cellular DNA (80,000 cell equivalents) from B lymphoblasts were used. To assess the sensitivity of the assay, 4, 0.4, 0.04, or 0.004 ng of DNA (800, 80, 8 and 0.8 cell equivalents) from the EBV lymphoblastoid cell line BXO1 was added.

### Cytogenetic analysis

Chromosome preparations of CD40-stimulated B cell lines at various times of culture were analyzed by standard G banding, or by 24-color multiplex FISH as described [51].

### Stimulation of B cells with oligonucleotides

Seven days after the last stimulation with CD40L and IL-4, B cells were harvested, washed twice, distributed to 48-well plates (0,25 million/0,5 ml/well) on top of a layer of irradiated CD40L-expressing or non-expressing murine L fibroblasts (20,000/well), and stimulated with phosphorothioate oligonucleotides at 4 µg/ml for 4 days. Supernatants were then harvested, and their content of IL-6 and IL-12p40 was assessed in standard ELISA assays according to the manufacturer's instructions (Mabtech, Nacka, Sweden).

We used the following phosphorothioate oligonucleotides: “CpG” (ODN 2006 [31], TCG TCG TTT TGT CGT TTT GTC GTT), “GpC” (ODN 2137 [33], TGC TGC TTT TGT GCT TTT GTG CTT), both purchased from Invivogen; and “CCC” (CCC CCC CCC CCC CCC CCC CC), synthesized by Metabion, Martinsried, Germany.

### Expansion of antigen-specific T cells

PBMC were purified from peripheral blood by standard Ficoll centrifugation and stimulated by repeated addition of irradiated CD40-stimulated B cells loaded with peptide. CD40-stimulated B cells were used 4 to 6 days after their last CD40 stimulation. For peptide loading, they were incubated with peptide in medium at 1 µg/ml for 1 h at 37°C, washed three times, and irradiated (50 Gy). They were then counted and used in PBMC stimulation. PBMCs were stimulated on day 0 at 2.5 mio/ml and at a PBMC∶B cell ratio of 10∶1. On days 9, 16, 23, and 30, PBMC-derived T cell cultures were stimulated at 2 mio/ml, with B cells added at a responder:B cell ratio of 4∶1. Interleukin-2 (“Proleukin”, Chiron) was added at 10 U/ml from day 9 and at 50 U/ml from day 16.

Antigenic peptides representing HLA-A2-restricted T cell epitopes were synthesized to 70% purity by JPT, Berlin, Germany. The peptides had the sequences NLVPMVATV (abbreviated NLV) derived from CMV pp65; GLCTLVAML (GLC) from the EBV BMLF1 gene product; CLGGLLTMV (CLG) from the EBV LMP2 protein; and ELAGIGILTV (ELA), a variant (A27L) of a peptide (amino acids 26-35) from MelanA/MART-1.

A calcein release assay [52] was used to assess antigen-specific cytotoxicity. Target cells (>1 million) were loaded with calcein acetoxymethylester (10 µg/ml; calcein AM, Invitrogen) in 1 ml medium for 30 minutes at 37°C, washed three times, and distributed to V-bottom 96-well plates at 5000 cells/100 µl/well. T cells were added in 100 µl/well in various concentrations. After 3 hours at 37°C, 150 µl of supernatant were transferred to flat-bottom 96-well plates. Fluorescence of released calcein was measured using fluorescein settings in a Wallac Victor plate reader (Perkin-Elmer). Target cell lysis was calculated relative to controls. Spontaneous fluorescent label release (no T cells added to targets) was interpreted as 0% lysis, maximal release (0.5% Triton X-100 added instead of T cells) as 100% lysis. Target cells included pp65-expressing and non-expressing mini-LCLs that had been established as described [36]. Melanoma cell lines A375 (ATCC no. CRL-1619) and Mel624.38 [53] were kindly provided by Dr. Elfriede Nößner, Munich, Germany.
